# Pulsed Light Application for *Campylobacter* Control on Poultry Meat and Its Effect on Colour and Volatile Profile

**DOI:** 10.3390/foods11182848

**Published:** 2022-09-14

**Authors:** Esther Baptista, Ana Borges, Teresa Aymerich, Susana P. Alves, Luís Telo da Gama, Helena Fernandes, Maria José Fernandes, Maria João Fraqueza

**Affiliations:** 1 CIISA—Centre for Interdisciplinary Research in Animal Health, AL4AnimalS—Associate Laboratory for Animal and Science, Faculty of Veterinary Medicine, University of Lisbon, Avenida da Universidade Técnica, Polo Universitário do Alto da Ajuda, 1300-477 Lisbon, Portugal; 2 IRTA—Institut de Recerca i Tecnologia Agroalimentàries, 17121 Monells, Spain

**Keywords:** *Campylobacter*, pulsed light, emergent technologies, poultry meat, safety

## Abstract

Campylobacter on poultry meat needs to be controlled to reduce the risk of infection caused by the consumption of chicken meat. Pulsed light (PL) application on poultry meat was studied to control *Campylobacter* spp. The effect of this technology was evaluated regarding poultry meat colour and volatile compound changes. Two breast sample groups were prepared: inoculated with *Campylobacter* (10^7^ bacteria of *Campylobacter jejuni* strains) and not inoculated. Samples were submitted to PL, five pulses/s of 300 ms, 1 Hz, and 1 J/cm^2^ in the apparatus, PL Tecum unit (Claranor). A response surface experimental design was applied regarding the factors of voltage (1828 to 3000 W) and distance to the source UV lamp (2.6 to 5.4 cm). The binomial factorial treatment (voltage and distance) with PL induced different energy doses (fluence J/cm^2^) received by samples, 2.82 to 9.67 J/cm^2^. Poultry meat pulsed light treated had a significant decrease of *Enterobacteriaceae* counts. The treatments applied were unable to reduce 1 log *Campylobacter* cfu/g of poultry meat. The poultry meat PL treated became slightly light, redder, and yellower than those not treated. PL can decrease the proportion of aldehydes on total volatiles in meat, particularly on those associated with chicken-like, chicken skin-like, and sweet odour notes in fresh poultry meat. Further studies of PL with higher energy doses will be necessary to confirm if there are *Campylobacter* reductions and about poultry meat treated under storage to evaluate if volatile compounds can affect the flavour of PL-treated meat samples.

## 1. Introduction

Worldwide, Campylobacteriosis is one of the most reported foodborne diseases. It is known that the real impact of this foodborne disease on human health stands underestimated because of the enormous economic losses due to health costs [1,2]. *Campylobacter jejuni* and *Campylobacter coli* are the main species involved in these reported cases. Poultry was identified as the main reservoir of these pathogens, being associated with more than 80% of human infections. Over the last decade, considerable efforts have been made to control *Campylobacter* spp., and potential interventions to control the agent throughout the poultry meat production chain have been indicated [1,3]. In Europe, since 1st January 2018, a new hygiene criterion regarding *Campylobacter* in poultry carcasses has been introduced by Regulation (E.C.) No. 2017/1495 [4]. The criterion defines a limit of *Campylobacter* <1000 cfu/g in chicken carcasses, being relevant to its accomplishment of minimizing the occurrence of campylobacteriosis outbreaks attributed to the consumption of poultry meat. By 2025 the level of safety will be increased to only 10 from 50 carcasses that could be over the criterion established. From all these facts, the industry is trying to understand the best intervention to implement and control *Campylobacter* in poultry meat. It was estimated that a reduction of more than 50% in the risk of infection caused by consumption of chicken meat could be achieved if the carcasses respect the limit of 1000 cfu/g [3]. Quantitative microbial risk assessments indicated that even moderate reductions in the numbers of *Campylobacter* on carcasses (>1 log10 per carcass) could significantly reduce the risk of infection in humans with a substantial decline in the incidence of Campylobacteriosis [1,3,5].

Many interventions have been hypothesized and studied at the poultry production and processing level to control *Campylobacter* [3,6,7,8,9,10]. Regarding interventions at the processing level, while chemical decontamination (lactic acid, chlorine dioxide, acidified sodium chlorite, and trisodium phosphate) of poultry carcasses was permitted in the USA, this method was not allowed in the European community [3]. Only physical technologies, such as freezing, crust-freezing, cooking, hot stream, high hydrostatic pressure, and ultrasounds, can be applied for decontaminating poultry carcasses [3,11,12]; nevertheless, their applications have limitations regarding efficiency, cost, and consumer acceptability. From EFSA [3], UV light was highlighted as a possible strategy at the processing level to control *Campylobacter* on carcasses; however, the strategy’s efficacy at the industrial scale was not concluded. In the USA, this technology gained approval by the Food and Drug Administration (FDA) since 1996 for food-surface disinfection applications [13]; yet, it still has not been used on a large scale by industry.

Pulsed-light (PL) processing is gaining popularity in food processing. PL technology is a non-thermal technology where sterilization and decontamination are achieved by using high-intensity light pulses of short duration on surfaces of foods, improving food safety and stability and not causing the damage produced by heating [14]. PL mechanisms to inactivate microorganisms are due to (i) a photochemical action based on the inhibition of new DNA chains during DNA replication due to the formation of dimers; (ii) to photothermal action due to the increase of temperature; and iii) to photophysical damage of cell membrane and elution of protein [15]. However, distinct sensitivities have been described in bacteria species and on strain level [15,16].

According to Mc Leod et al. [17], the exposure of the raw chicken fillet surface to various doses of UV-C (fluences from 0.05 to 3.0 J/cm^2^;10 mW/cm^2^, from 5 to 300 s) or pulsed UV (fluences from 1.25 to 18.0 J/cm^2^) represents practical alternatives for reducing the viability of pathogenic and spoilage bacteria on this product. Haughton et al. [18] described in their study that inoculated raw skinless chicken fillet treated with UV light up to 0.192 J/cm^2^ had significant (*p* < 0.05) reductions of 0.76, 0.98, 1.34, 1.76, and 1.29 log CFU/g for *C. jejuni*, *E. coli*, *Salmonella* Enteritidis, total viable counts, and *Enterobacteriaceae*, respectively. Additionally, Cassar et al. [19] studied the effectiveness of pulsed ultraviolet light for the destruction of *Salmonella*, *E. coli*, and *Campylobacter* on the surface of chicken thigh meat, and on the thigh skin surfaces, the variables of study are the distance (8 and 13 cm) and treatment time (0, 5, 15, 30, and 45 s). The authors concluded that pulsed UV light exposure for 5 and 45 s on thigh skin surfaces resulted in log10 reductions of 1.19 and 1.96 for *E. coli*; 1.08 and 1.85 for *Campylobacter*; and 0.90 and 1.82 for *Salmonella*, respectively. The exposure to PUV light for 5 and 45 s on lean surface thighs resulted in higher log_10_ CFU/cm^2^ reductions (1.22 and 2.02 for *E. coli*, 1.45 and 2.09 for *Campylobacter*, and 1.55 and 2.42 for *Salmonella*, respectively).

Poultry meat can be potentially contaminated on its surface by *Campylobacter* due to its capability to easily adhere [20], mainly during slaughtering and deboning practices. The objective of this study was to assess the effect of pulsed light (including UV, representing 10–15% of total spectra) treatments using a central composite rotatable design (CCRD) with a binomial combination of the variables, voltage and distance to the source of the pulsed light lamp, with the same number of pulses, responsible for the production of total energy delivered to the sample (fluence, J/cm^2^). We intend to choose the best variable combination that could reduce the contamination of *Campylobacter jejuni* on poultry meat with less impact on colour and also on those volatile aldehydes related to meat flavour.

## 2. Materials and Methods

### 2.1. Strains and Preparation of Bacterial Suspensions for Inoculation

Three different strains of *Campylobacter jejuni* were used in this experiment (*Campylobacter jejuni* NCTC 11,168 and two wild strains, *C. jejuni* 4 isolated from poultry wings, and *C. jejuni* A3 (1), isolated from poultry cecum content). These bacteria were cultured in Columbia agar (BioMérieux, Caponne, France) at 42 °C under microaerophilic conditions (Genbox Microaer, BioMérieux, Caponne, France). An initial culture suspension was prepared for each strain in NaCl 0.9%, with an optical density adjusted to an OD600 of 0.4–0.5. Serial dilutions of these strain suspensions were plated on CampyFood agar (BioMérieux, Caponne, France) and estimated at approximately 8–9 log *Campylobacter*/mL. A mixed bacterial suspension was done with an equivalent proportion of these three strains on every sample inoculated with 50 µL of the suspension to obtain approximately 5 log cfu/g.

### 2.2. Poultry Meat Sample Preparation

Poultry meat breasts were purchased at a local supermarket, frozen, and stored at -20 °C for 48h to eliminate initial contamination of *Campylobacter*. The breasts without skin were defrosted at 4 °C, then cut in squares 5 × 3 cm with 4–5mm thickness, and two groups of samples were prepared: one inoculated with 5 log cfu/g of a pool of the three *Campylobacter jejuni* strains and the other not inoculated. All samples were vacuum packaged (vacuum-packer EV-15-2-CD, Tecnotrip, Terrassa, Spain) with low-strength vacuum pack polyamide and polypropylene (PA/PP 90) bags (O_2_ permeability: <70 cm^3^/m^2^/day and 0% RH; CO_2_ permeability: <350 cm^3^/m^2^/day and 75% RH; N_2_ permeability: <17 cm^3^/m^2^/day and 75% RH; water-vapor permeability: <4.5 g/m^2^/day at 23 °C and 85% RH; SISTEMCOC-IN, Sistemes d’Embalage ESTUDI GRAF, S.A., Aiguaviva, Girona, Spain) to simulate microaerophilic conditions being submitted to pulsed-light treatments. The group of samples not inoculated were treated and used for microbial and physical–chemical determinations; the inoculated group samples were treated and used only for *Campylobacter* spp. counts.

### 2.3. Pulsed-Light Treatments

Pulsed-light conditions considering electric potential difference (volt, V) and distance (D, cm) of the source of pulsed light to the sample with fixed five pulses were investigated by the response surface methodology (RSM) used for modelling and optimization of multiple variables to predict the best performance conditions with a minimum number of experiments [19]. The experiments were carried out with a central composite rotatable design (CCRD) as a function of electric potential difference (voltage) and distance to the source of the pulsed-light lamp (D) with five levels for each factor, which allowed fit of first- or second-order polynomials to the experimental data points [21]. Twelve experiments were carried out testing different voltages (ranging from 1828 to 3000 V) and distance to the source of the pulsed-light lamp (ranging from 2.6 to 5 cm), considering four factorial points resulting from combinations of levels coded as (+1) and (−1) for both voltage and D; four-star points coded as +√2 and −√2 for combinations of voltage and D, and three centre points coded as 0 (Table 1). A group not submitted to pulsed-light treatment was used as a control. For each binomial combination, replications (n = 3) were carried out.

According to the experimental design, all samples were submitted to pulsed light with fixed factor and number of pulses (five pulses of 300 µs/each) and frequency (1 Hz). The equipment for high-intensity light pulses (PL) was a PL Tecum Unit (Claranor, Manosque, France) with high frequencies of 15% UVC light (200–280 nm), 50% visible light (280–780nm), and 35% proximal IR light (780–1100 nm). The distance between the sample and the quartz window of the PL lamp could be changed by moving the sample rack up or down. The fluence (J/cm^2^) received by samples was measured by a laser power detector (JoulmeterUP17P connected to MAESTRO-monitor, Gentec-EO, Quebec city, Canada). Immediately after treatment, all samples were analyzed. Subsequently, samples for volatile aldehydes were kept under −80 °C until the analyses.

### 2.4. Microbial Analysis

The preparation of samples, initial suspension, and decimal dilutions for microbiological analyses were performed according to [22]. Microbial determinations were carried out for total psychrotrophic count (PCA, Scharlau) after incubation at 10 °C for five days, *Enterobacteriaceae* count (Violet Red Bile Dextrose agar, Scharlau Chemie, Spain) after incubation at 37 °C for two days [23]; and thermophilic *Campylobacter* spp. count (Campyfoods, Biomerieux, France) after incubation at 42 °C for 48 h [24]. All counts were expressed as log cfu/g.

### 2.5. Physicochemical Analysis

*Temperature determination*. Before and after pulsed-light treatments, the meat temperature was measured by an IR-thermometer (Testo, Barcelona, Spain). The sample temperature variances were calculated. The average of three determinations was retained for further data analyses.

*Colour evaluation*. Meat colour measures using the coordinates L*, a*, and b* of the CIELAB colour system just before the package opening were performed with a colourimeter (Minolta CR-300, Chromometer, Osaka, Japan). A white tile was used to calibrate the instrument according to the manufacturer’s instructions. The average value of the three, L*, a*, and b* measurements, was used for statistical analysis.

*Volatile aldehydes*. The volatile aldehydes in the meat samples were analyzed by solid-phase microextraction (SPME) and gas chromatography-mass spectrometry (GC-MS) adapted from [25]. Briefly, about 5 g of meat sample was minced, weighted to a glass vial, and closed with an aluminium cap with a PTFE-septum. Samples were then conditioned at 35 °C for 15 min, and afterwards, a divinylbenzene/carboxen/polydimethylsiloxane (DVB/Carboxen/PDMS) Stable Flex SPME fiber (50/30 μm; 2-cm long) (Supelco, Bellefonte, PA, USA) was exposed to headspace for 30 min at 35 °C. The fibre was inserted in the injector of the Shimadzu GC-MS QP2010 Plus (Shimadzu, Kyoto, Japan), set at 250 °C, and kept for 30 min to complete the fibre desorption.

The GC-MS had an SPB-5 capillary column (30 m × 0.25 mm internal diameter × 0.25 μm film thickness, Supelco Inc., Bellefonte, PA, USA), and the initial oven temperature was set at 40 °C, held for 8 min, and then increased to 220 °C at 4 °C/min and held for 20 min. Helium was used as the carrier gas at a 1 mL/min flow rate. The MS conditions were as follows: ion source temperature, 220 °C; interface temperature, 220 °C; ionization energy, 70 eV; scan, 35–500 atomic mass units. Before each analysis, the SPME fiber was conditioned for 30 min at 250 °C in the GC injector. The identification of the aldehydes was performed by comparison with the mass spectra of the NIST/EPA/NIH Mass Spectral Database (Version 2008), by comparing with commercial standards and using the linear retention index (LRI) that was calculated using the retention times of a homologous series of n-alkanes C5–C25. Aldehydes were expressed as a percentage of total volatile compounds in the chromatograms.

### 2.6. Statistical Analysis

Statistical analyses were performed with the SAS software package, version 9.4 (SAS Institute, Cary, NC, USA). The RSREG procedure was used, including linear and quadratic effects of the two variables under study (voltage (V) and distance (D)), as well as the interaction between their linear effects in the linear model where the response variables were the microbiological counts and physicochemical parameters assessed. A surface described by a second-order polynomial equation was fitted to each set of experimental data points. Additionally, the linear and quadratic effects of the irradiance dose fluence (J/cm^2^) were considered.

In addition, the GLM procedure of SAS was used to perform the analyses of variance by comparing the results obtained with the treated vs. untreated samples and then the results from the various treatment combinations with each other. The mean ± standard error (SE) of microbiological counts and physicochemical parameters for the various binomial combinations of V and D were obtained, and the differences between the treatments were tested using Tukey’s post hoc test.

## 3. Results and Discussion

### 3.1. Pulsed-Light Treatment: Energy Irradiation Received on Samples

**Figure 1:**
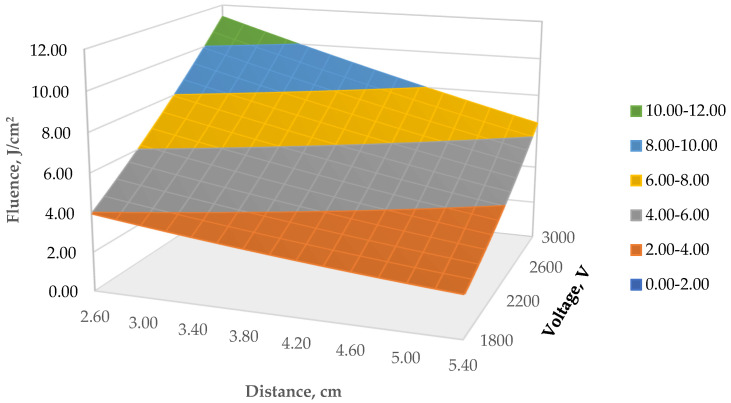
Response surface of binomial factorial electric potential difference (volt, V) and distance (D, cm) of the light source over fluence (J/cm^2^).

The binomial treatment with an electric potential difference ranging from 1828 to 3000 V and distance to the source UV lamp (ranging from 2.6 to 5 cm) had different energy irradiation rates on the samples, as described by other authors [26], measured as the radiant energy that is received on sample surfaces per unit area and named fluence (J/cm^2^) (Figure 1). This means that the energy dose (fluence J/cm^2^) given to the poultry samples varied from 2.82 ± 0.06 (J/cm^2^) to 9.68 ± 0.15 (J/cm^2^). According to the model, with the same number of pulses (five) a higher voltage gives higher fluence values, while lower fluence values were observed for higher distances from the source light. Additionally, Hsu and Moraru [26] have stated that fluence decays exponentially with increasing vertical distance from the lamp.

**Figure 2:**
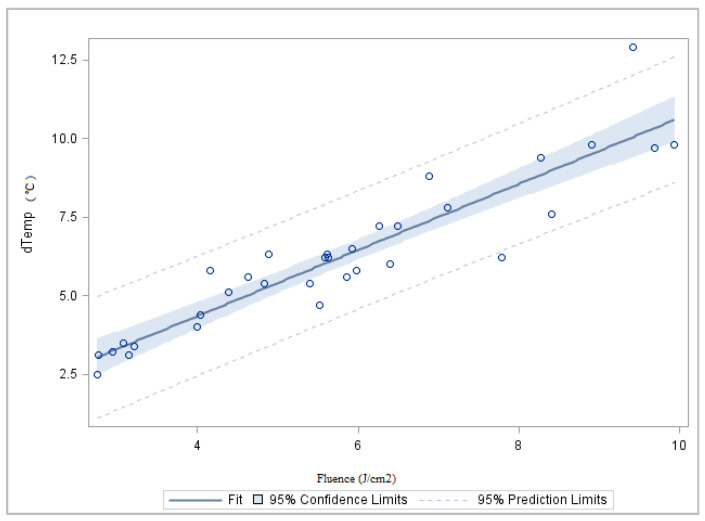
Effect of the PL energy dose on samples temperature (°C).

Based on the dose of energy received by the poultry meat samples, it was possible to observe an increase in their temperature from 2.5 °C to 10 °C according to increased doses of fluence (Figure 2). Other authors also reported this significant temperature increase when fluence increased [16,27].

### 3.2. Effect of Pulsed-Light Treatment on Spoilage Microorganisms and Campylobacter

#### 3.2.1. Spoilage Microorganisms

Poultry meat samples presented counts of 3.1 ± 0.3 log cfu/g and 2.5 ± 0.3 log cfu/g for total psychotropic and Enterobacteriaceae, respectively (see Appendix A Table A1). As described by other authors [28,29], this initial level of contamination was typical on poultry meat for these microbial groups. In fact, and according to Nieminen et al. [29], the microbiota of poultry meat is composed 54.1% by sequences assigned mainly to Gram-negative bacteria families, with 17.4% belonging to the *Enterobacteriaceae* family, while the majority 32.9% was represented by *Vibrionaceae* with references to *Shewanella* and *Aeromonas*. *Pseudomonas* spp., *Acinetobacter* spp., and *Flavobacterium* spp. were also common in aerobically packaged poultry meat. Gram-positive bacteria, *Brochotrix*, *Carnobacterium*, *Lactobacillus*, *Lactococcus*, and *Leuconostoc* genera, can grow under refrigeration temperatures and have an important role in the spoilage of poultry meat under modified atmosphere package.

**Figure 3:**
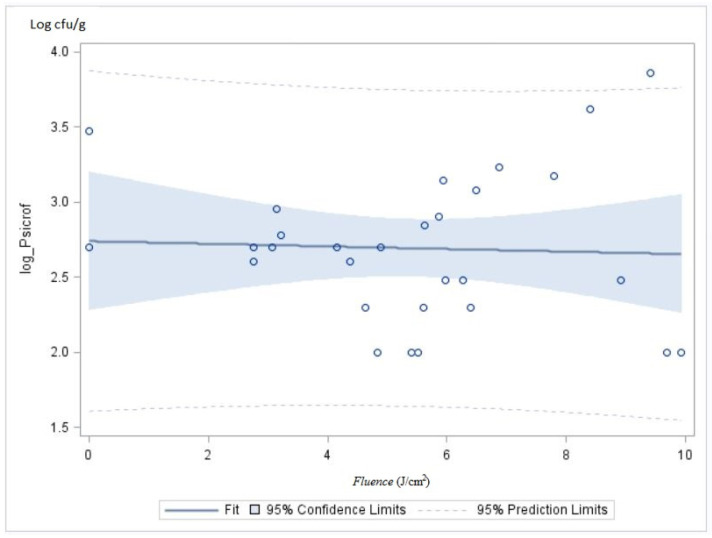
Effect of PL treatment energy dose on psychrothropic counts.

**Figure 4:**
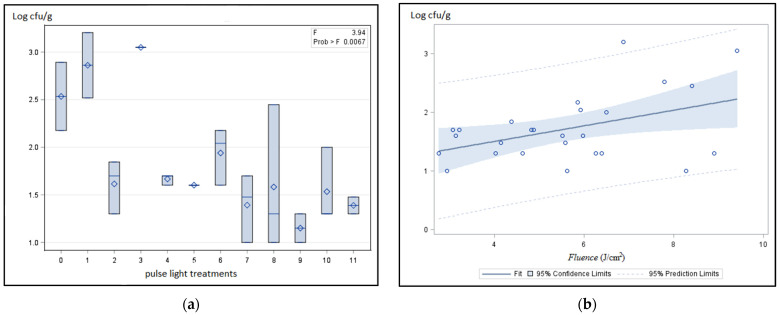
Effect of PL treatment over *Enterobacteriaceae* counts (**a**, blue shadow = dispersion of data /blue square = average, “–” = median) and according to fluence (J/cm^2^) (**b**).

In this study, the application of PL on samples did not promote any significant difference on total psychotropic counts independently of the increased energy dose applied (Figure 3). This could be related to the presence of psychotropic Gram-negative and positive bacteria, as described by [28,29], with different resistances to the PL treatments. PL inactivation of microorganisms was attributed to photothermal (thermal effect due to the increase of temperature) and/or photochemical (inhibiting formation of new DNA chains in the process of DNA replication due to the formation of dimers) and/or photophysical (damage of cell membrane and elution of protein) mechanisms that can affect more Gram-negative than Gram-positive bacteria [15,30,31]. In addition, the roughness of the matrix food could produce different shadows, which contribute to differences in bacteria inactivation [13,16,19]. After all, PL was more effective regarding Gram-negative bacteria since the samples treated by PL had a decrease of *Enterobacteriaceae* counts when compared to samples not treated, approximately 1–1.3 log cfu/g (Figure 4a).

This data was interesting since with low fluence (lower than 5 J/cm^2^) there was a count reduction induced by a photochemical action. This reduction of Enterobacteriaceae counts, a hygiene indicator, can be indirectly related to the control and reduction of spoilage bacteria and potential pathogens. Chintagari et al. [32] in their studies in vitro, observed reductions of *E. coli* under pulsed UV light with a range of 2.4–9.6 J/cm^2^. Additionally, other authors [19,33] reported a reduction of *Salmonella* and *E. coli* on poultry meat but not more than 2 log cfu/g when pulsed UV light was applied at 8 cm with exposure time of 45 s or at 5 cm during 15 s for unpackaged samples and 5 cm-30 s for vacuum-packaged samples.

However, and surprisingly, increased doses of fluence (J/cm^2^) among treated samples (treatments 1 and 3) did not decrease the *Enterobacteriaceae* counts (Figure 4b) significantly. The photothermal action with a temperature increase to 10 °C induced by higher energy doses (fluence, J/cm^2^; Figure 2) did not promote a higher bactericidal effect. This *Enterobacteriaceae* family that includes mesophilic bacteria was not significantly affected under higher energy doses and temperature.

#### 3.2.2. *Campylobacter*

**Figure 5:**
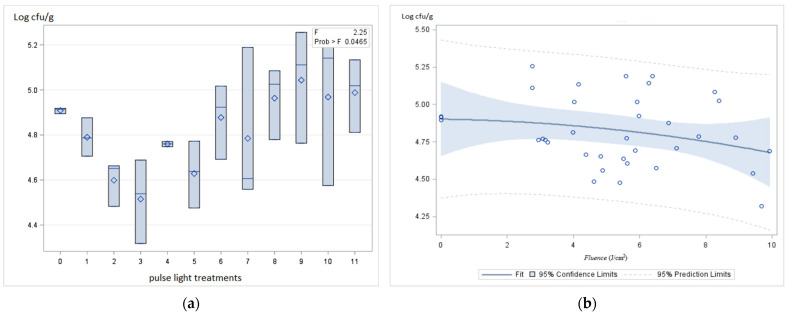
Effect of PL treatment over *Campylobacter jejuni* counts (**a**, blue shadow = dispersion of data /blue square = average, “–“ = median) and according to fluence (J/cm^2^) (**b**).

The samples inoculated with *Campylobacter jejuni* presented counts of 4.9 ± 0.01 log cfu/g (Table A1). On treatment 3 (3 cm and 2828 V) with a higher energy dose of 9.68 ± 0.15 J/cm^2^, the *Campylobacter* counts were significantly lower (4.5 ± 0.01 log cfu/g, *p* < 0.05). However, this slight reduction inferior to 1 log shall not have an impact on *Campylobacter* control on poultry meat. Overall, the counts on poultry meat samples treated with PL were not significantly different when compared to those of contaminated samples not treated (Figure 5a). According to the model presented in Figure 5b, when the fluence increases, there is not a significant effect on *Campylobacter* counts.

While for Gram-negative group *Enterobacteriaceae*, the pulsed-light photochemical and/or photophysical (without photothermal influence) actions can be the main mechanisms that affected them with a significant count decrease. For *Campylobacter* bacteria, it seems that it was mainly the photothermal action with a respective increase of temperature that induced some bacteria lethality when the fluence was higher. PL will be more effective only when a concomitant temperature increases, inducing a higher denaturation of essential proteins and enzymes. Cassar et al. [19] reported that for the same fluence, the intensity of the energy related to the distance of the sample to the source of light could influence the antimicrobial action of pulse light. In addition, in a study with higher intensities of near-ultraviolet/visible (NUV–vis) light, it was reported that the combination of more extended treatment times (5 min) and reduced distance (3 cm) resulted in significant temperature increases (over 65 °C) with the greatest reductions for *C. jejuni* (2.62 log_10_ cfu/g on the raw skinless chicken fillet) [34]. *Campylobacter* spp. Is a Gram-negative bacteria, but due perhaps to their helical-shaped and protection of the matrix (poultry meat) [13,14,35], they have been more resistant than other Gram-negative species to irreversible cell damage and molecular (cytotoxic effects in the cell, including cell membrane damage, rupture of the plasma membrane, DNA adduct formation, metabolic damage) induced by PL treatment [11]. The shadowing effect of the raw skinless breast poultry cuts could be the main reason for the PL treatment, even under short distances and high fluences, and do not have an impact on *Campylobacter* reduction [11,14,17].

### 3.3. Effect of Pulsed Light Treatment on Poultry Meat Colour and Volatile Aldehydes Profile

At the purchase moment, meat colour is the main characteristic influencing consumer choice [36]. Several factors causing oxidation can influence meat colour with subsequent discolouration [37,38,39]. Poultry meat has a lighter pink colour than other meats. The results of the colour assessment on samples PL treated are presented in Table A1. The poultry meat colour presented an L* value of 48.84 ± 1.08; a* value of 3.76 ± 0.98, and b* value of 22.40 ± 1.95. These values of L* and a* were similar and in the range observed for breast meat by [40]; only the b* were higher, denoting these samples a higher yellow colour.

**Figure 6:**
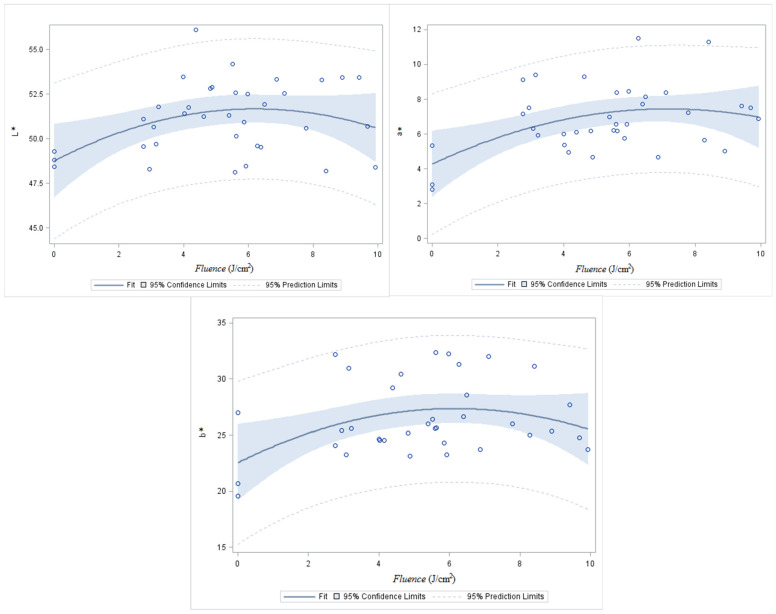
Effect of PL over meat color L*a*b* parameters.

After PL treatments (Figure 6), the poultry meat samples became slightly lighter, redder, and yellower than meat not treated. The PL treatment did not influence colour parameters, L* and b*, when the irradiance increased, and only a* was significantly influenced.

Additionally, authors reported that chicken breasts exposed to high doses of UV light were shown to be redder with a slight increasing of yellow colouration [41]. However contrary to what was obtained in this study, the chicken breasts were slightly darker but without statistical significance. The meat colour is dependent on the myoglobin oxidation state. The L* value remains constant during pigment meat oxygenation and oxidation [42]. Nevertheless, the muscle proteins could be oxidized, and the photothermal action of UV light could change their conformation [43], which could explain a higher reflection of the light and increased values of L*. Usually, the oxidation of myoglobin in meat is translated to a decrease of a* and b* values [42]; however, in this study, we had a significant increase of a* in poultry samples PL treated; this observation was also supported by Park and Ha [41]. Moreover, an increase of a* was described in irradiated poultry meat by Xiao et al. [44].

Lipid oxidation was pointed out as the primary cause of flavour deterioration and development of “warmed-over flavour (WOF)” in poultry meat, known as oxidized flavours. Usually, storage and cooking are the main factors inducing the formation of WOF. The application of UV light on meat was mentioned as a pro-oxidant factor developing off-flavours due to photochemical effects on the lipid fractions of the meat or caused by the absorption of ozone and oxides of nitrogen [45]. Some aldehydes are lipid oxidation products in poultry flavour [46,47]; thus, changes in the aldehyde profile of PL-treated poultry samples could result from lipid oxidation.

In the raw broiler meat samples (Table 2), initially identified as the main components from a relative total proportion of volatile aldehydes (23.64% ± 4.26%) were the: Hexadecanal (6.55% ± 0.78%), Nonanal (6.47% ± 1.69%), Benzaldehyde (2.54% ± 0.31%), (E, E)- 2,4- Decadienal (1.45% ± 0.19%), and Hexanal (1.06 ± 1.74%). Other volatile aldehydes were identified in smaller proportions, such as Heptanal, Octadecanal, and Octanal (0.71%-0.65%). The poultry samples presented low amounts of aldehydes, as described by [48], since they are low-fat raw meat. This study’s main aldehydes, presented on raw poultry samples, are expected to derive from MUFA and PUFA oxidation, hydrolysis of phospholipids (plasmalogens), and Strecker degradation [49,50,51]. Nonanal has a low odour threshold value and can be associated with lipid oxidation of oleate hydroperoxide primary products with green and fatty descriptions [50]. (E, E)-2,4-decadienal (C26) is considered to be the most important odorant for chicken flavour compared to hexanal due to its much lower odour threshold [47]. Pentanal, hexanal, and (E, E)-2,4-decadienal were primarily responsible for the WOF in meat, which caused a loss of meaty, chicken-like, and sweet odour notes and the formation of green, cardboard-like, and metallic off-odours [52]. Hexanal and 2,4-decadienal are the most abundant aldehydes identified in chicken flavour, known to be linoleic acid’s primary oxidation products. Long-chain aldehydes as the hexadecanal and octadecanal might derive from the hydrolysis of phospholipids, particularly plasmalogens, in poultry meat [53,54]. Plasmalogen contains a vinyl ether substituent with 16:0 or 18:0 alkyl groups at the sn-1 position of glycerol, which generates the corresponding aldehydes on hydrolysis. The presence of hexadecanal and octadecanal have been reported in raw or heated meat from several species, including poultry [49,53,54] and in the chicken breast meat [55]. Benzaldehyde was also detected in poultry meat samples, and it is a crucial aroma component existent in the breast and generated by the Strecker reaction of some amino acids, such as leucine and phenylalanine [52].

In general, and independently of the binomial conditions of this study, the treatments with PL can induce significant differences in poultry meat flavour. Indeed, compared to the control, a relative reduction of all aldehydes in PL-treated poultry samples was observed. It seems that PL could induce the loss of chicken-like, chicken skin-like, and sweet odor notes in fresh poultry meat with the significant reduction of hexadecanal, nonanal, benzaldehyde, (E, E)-2,4-decadienal (1.45% ± 0.19%), and hexanal apart from all other aldehydes. The relative reduction of these aldehydes in total meat volatiles suggests that they might be sensitive to PL, resulting in the formation of other volatiles compounds. In fact, aldehydes and ketones were reported to be susceptible to photochemical reactions through Norrish-type reactions, resulting in the formation of other derivatives [56,57]. Further studies will be needed to evaluate the photochemical products that might be generated from aldehydes in pulsed-light-treated meat under storage. Despite that, evident prooxidant action and the development of lipid oxidation with the application of PL were not expected because samples were from the breast with low-fat content, all samples were vacuum packaged, and the application of PL did not increase sample temperature relevantly. Moreover, the high total antioxidant capacity (TAC) and very low Mb content in chicken breast described by Min et al. [58] might have a protective effect due to a diet supplemented with vitamin E [48].

## 4. Conclusions

Poultry meat pulsed light treated had a significant decrease of *Enterobacteriaceae* counts, approximately 1–1.3 log cfu/g. This intervention could help control the presence of *Enterobacteriaceae* or potential pathogens from this family on poultry meat.

The counts of *Campylobacter* were significantly lower (4.5 log cfu/g) on treatment 3 with a higher fluence energy dose when a short distance (3 cm) and higher voltage (2828 V) were applied. However, though statistically significant, the reduction observed does not impact *Campylobacter* control since it was less than 1 log cfu/g. Further studies will be necessary with PL and higher energy doses on poultry meat to confirm a reduction of *Campylobacter*.

After PL treatments, the poultry samples became slightly light, redder, and yellower than samples not treated. In general, and independently of the binomial conditions and energy dose applied, the PL can induce significant differences in poultry meat with a relative reduction of aldehydes. Nevertheless, other volatile compounds might have been generated from the photochemical reaction of raw meat aldehydes under PL Thus in the future, further studies of meat under storage will be needed to evaluate if those volatile compounds can affect the flavour of PL-treated meat samples.

## Figures and Tables

**Table 1:** Experimental data obtained for the optimization of voltage (V) and distance (cm) to the light source applied to poultry samples.

No	Voltage/V	Distance/cm
Control	0	0
1	2414	2.6
2	2000	3
3	2828	3
4	1828	4
5	2414	4
6	2414	4
7	2414	4
8	3000	4
9	2000	5
10	2828	5
11	2414	5.4

**Table 2:** Volatile aldehydes (% of total volatile) of PL-treated poultry samples.

Treatment	Voltage (V)	Distance (cm)	Fluence (mJ/cm^2^)	Hexanal %	Heptanal %	Benzaldehyde %	Octanal %	Nonanal %	(E,E)-2,4-Decadienal %	Hexadecanal %	Octadecanal %	Other Aldehydes %	Total Aldehydes %
Control	0	0	0	1.06 ± 1.74 ^ab^	0.71 ± 0.23 ^a^	2.54 ± 0.30 ^a^	0.65 ± 0.18 ^a^	6.47 ± 1.69 ^a^	1.45 ± 0.19 ^a^	6.55 ± 0.78 ^a^	0.69 ± 0.19 ^a^	3.52 ± 0.82 ^a^	23.64 ± 4.26 ^a^
1	2414.25	2.6	7.26 ± 0.27	0.85 ± 1.74 ^ab^	0.00 ± 0.23 ^b^	0.26 ± 0.30 ^b^	0.25 ± 0.18 ^ab^	3.08 ± 1.69 ^ab^	0.00 ± 0.19 ^b^	0.00 ± 0.78 ^b^	0.00 ± 0.19 ^b^	0.00 ± 0.82 ^b^	4.44 ± 4.26 ^b^
2	2000.00	3.0	4.61 ± 0.13	5.76 ± 1.74 ^a^	0.00 ± 0.23 ^b^	0.00 ± 0.30 ^b^	0.34 ± 0.18 ^ab^	1.73 ± 1.69 ^ab^	0.00 ± 0.19 ^b^	0.00 ± 0.78 ^b^	0.00 ± 0.19 ^b^	0.00 ± 0.82 ^b^	7.82 ± 4.26 ^b^
3	2828.50	3.0	9.68 ± 0.15	0.00 ± 1.74 ^b^	0.00 ± 0.23 ^b^	0.00 ± 0.30 ^b^	0.08 ± 0.18 ^b^	1.05 ± 1.69 ^b^	0.00 ± 0.19 ^b^	0.00 ± 0.78 ^b^	0.00 ± 0.19 ^b^	0.00 ± 0.82 ^b^	1.13 ± 4.26 ^b^
4	1828.41	4.0	3.15 ± 0.04	0.00 ± 1.74 ^b^	0.00 ± 0.23 ^b^	0.22 ± 0.30 ^b^	0.20 ± 0.18 ^ab^	1.50 ± 1.69 ^b^	0.00 ± 0.19 ^b^	0.00 ± 0.78 ^b^	0.00 ± 0.19 ^b^	0.00 ± 0.82 ^b^	1.91 ± 4.26 ^b^
5	2414.25	4.0	5.51 ± 0.06	0.38 ± 1.74 ^b^	0.26 ± 0.23 ^ab^	0.59 ± 0.30 ^b^	0.30 ± 0.18 ^ab^	4.50 ± 1.69 ^ab^	0.00 ± 0.19 ^b^	0.00 ± 0.78 ^b^	0.00 ± 0.19 ^b^	0.00 ± 0.82 ^b^	6.02 ± 4.26 ^b^
6	2414.25	4.0	5.92 ± 0.03	0.00 ± 1.74 ^b^	0.00 ± 0.23 ^b^	0.00 ± 0.30 ^b^	0.04 ± 0.18 ^b^	2.80 ± 1.69 ^ab^	0.00 ± 0.19 ^b^	0.00 ± 0.78 ^b^	0.00 ± 0.19 ^b^	0.00 ± 0.82 ^b^	2.83 ± 4.26 ^b^
7	2414.25	4.0	5.37 ± 0.24	0.00 ± 1.74 ^b^	0.00 ± 0.23 ^b^	0.00 ± 0.30 ^b^	0.39 ± 0.18 ^ab^	3.92 ± 1.69 ^ab^	0.00 ± 0.19 ^b^	0.00 ± 0.78 ^b^	0.00 ± 0.19 ^b^	0.00 ± 0.82 ^b^	4.31 ± 4.26 ^b^
8	3000.09	4.0	8.53 ± 0.19	0.81 ± 1.74 ^ab^	0.21 ± 0.23 ^ab^	0.38 ± 0.30 ^b^	0.17 ± 0.18 ^ab^	5.21 ± 1.69 ^ab^	0.00 ± 0.19 ^b^	0.00 ± 0.78 ^b^	0.00 ± 0.19 ^b^	0.00 ± 0.82 ^b^	7.23 ± 4.26 ^b^
9	2000.00	5.0	2.82 ± 0.06	0.00 ± 1.74 ^b^	0.00 ± 0.23 ^b^	0.00 ± 0.30 ^b^	0.43 ± 0.18 ^ab^	4.31 ± 1.69 ^ab^	0.00 ± 0.19 ^b^	0.00 ± 0.78 ^b^	0.00 ± 0.19 ^b^	0.00 ± 0.82 ^b^	4.74 ± 4.26 ^b^
10	2828.50	5.0	6.38 ± 0.06	0.99 ± 1.74 ^ab^	0.00 ± 0.23 ^b^	0.00 ± 0.30 ^b^	0.00 ± 0.18 ^b^	2.43 ± 1.69 ^ab^	0.00 ± 0.19 ^b^	0.00 ± 0.78 ^b^	0.00 ± 0.19 ^b^	0.00 ± 0.82 ^b^	3.42 ± 4.26 ^b^
11	2414.25	5.4	4.06 ± 0.05	0.65 ± 1.74 ^b^	0.00 ± 0.23 ^b^	0.00 ± 0.30 ^b^	0.00 ± 0.18 ^b^	4.55 ± 1.69 ^ab^	0.00 ± 0.19 ^b^	0.00 ± 0.78 ^b^	0.00 ± 0.19 ^b^	0.00 ± 0.82 ^b^	5.20 ± 4.26 ^b^
Sig.	-	-	-	*	**	***	*	***	***	***	**	***	***

^ab^ within columns with the same letter are not significantly different. Sig. * = *p* < 0.05; ** = *p* < 0.01; *** = *p* < 0.001.

## Data Availability

Data is contained within the article.

## Appendix A

**Table A1:** Microbial and color parameter of PL-treated poultry samples.

Treatment	Voltage (V)	Distance (cm)	Fluence (J/cm^2^)	*Campylobacter* Log cfu/g	*Enterobacteriaceae* Log cfu/g	Psycrotrophic Log cfu/g	L*	a*	b*
Control	0	0	0	4.9 ± 0.1 ^ab^	2.5 ± 0.3 ^ab^	3.1 ± 0.4	48.84 ± 1.08	3.76 ± 0.98 ^c^	22.40 ± 1.95
1	2414.25	2.6	7.26 ± 0.27	4.8 ± 0.1 ^abc^	2.9 ± 0.3 ^a^	3.2 ± 0.4	52.17 ± 1.08	6.76 ± 0.98 ^ab^	27.24 ± 1.95
2	2000.00	3.0	4.61 ± 0.13	4.6 ± 0.1 ^bc^	1.6 ± 0.3 ^bcd^	2.3 ± 0.3	53.39 ± 1.08	7.19 ± 0.98 ^ab^	28.28 ± 1.95
3	2828.50	3.0	9.68 ± 0.15	4.5 ± 0.1 ^c^	3.1 ± 0.3 ^a^	2.6 ± 0.3	50.85 ± 1.08	7.33 ± 0.98 ^ab^	25.40 ± 1.95
4	1828.41	4.0	3.15 ± 0.04	4.8 ± 0.1 ^abc^	1.7 ± 0.3 ^bcd^	2.8 ± 0.3	50.73 ± 1.08	7.22 ± 0.98 ^ab^	26.61 ± 1.95
5	2414.25	4.0	5.51 ± 0.06	4.6 ± 0.1 ^bc^	1.6 ± 0.3 ^abcd^	2.1 ± 0.3	52.69 ± 1.08	7.21 ± 0.98 ^ab^	28.27 ± 1.95
6	2414.25	4.0	5.92 ± 0.03	4.9 ± 0.1 ^ab^	1.9 ± 0.3 ^bc^	2.8 ± 0.3	50.65 ± 1.08	6.92 ± 0.98 ^ab^	26.60 ± 1.95
7	2414.25	4.0	5.37 ± 0.24	4.8 ± 0.1 ^abc^	1.4 ± 0.3 ^cd^	2.7 ± 0.4	50.39 ± 1.08	5.81 ± 0.98 ^bc^	24.81 ± 1.95
8	3000.09	4.0	8.53 ± 0.19	5.0 ± 0.1 ^a^	1.6 ± 0.3 ^cd^	3.1 ± 0.4	51.65 ± 1.08	7.32 ± 0.98 ^ab^	27.18 ± 1.95
9	2000.00	5.0	2.82 ± 0.06	5.0 ± 0.1 ^a^	1.2 ± 0.3 ^d^	2.7 ± 0.4	49.66 ± 1.08	7.94 ± 0.98 ^ab^	27.22 ± 1.95
10	2828.50	5.0	6.38 ± 0.06	5.0 ± 0.1 ^a^	1.5 ± 0.3 ^cd^	2.6 ± 0.3	50.34 ± 1.08	9.12 ± 0.98 ^a^	28.86 ± 1.95
11	2414.25	5.4	4.06 ± 0.05	5.0 ± 0.1 ^a^	1.4 ± 0.3 ^cd^	2.7 ± 0.5	52.22 ± 1.08	5.44 ± 0.98 ^bc^	24.58 ± 1.95
Sig.	-	-	-	*	**	NS	NS	*	NS

^abcd^ within columns with the same letter are not significantly different. Sig. NS = not significant, * = *p* < 0.05; ** = *p* < 0.01.
